# Association Between Public Insurance and Quality of Life Outcomes in Surgically Treated Adolescent Idiopathic Scoliosis

**DOI:** 10.7759/cureus.93776

**Published:** 2025-10-03

**Authors:** Emily D Ferreri, Kathryn R Segal, Anisha Duvvi, Zachariah Samuel, Mohamed Said, Leila Alvandi, Eric Fornari, Jamie A Gomez, Jacob Schulz

**Affiliations:** 1 Department of Orthopaedic Surgery, Division of Pediatric Orthopaedic Surgery, Montefiore Einstein, Bronx, USA

**Keywords:** adolescent idiopathic scoliosis, health disparity, insurance, posterior spinal fusion, quality of life

## Abstract

Background: The role of social determinants of health is becoming increasingly studied in orthopedic diseases. In adolescent idiopathic scoliosis (AIS), patients with public insurance have been shown to have larger Cobb angles, longer waits for surgery, and longer hospitalizations, although the literature is conflicting. We do not yet know how insurance type is associated with patient quality of life (QOL) in AIS patients who require surgical management.

Methods: This retrospective cohort study was conducted among AIS patients surgically managed with posterior spinal fusion (PSF). The primary independent variable was insurance type, classified as public or private. For patient-reported outcomes, we used the Scoliosis Research Society Patient Outcome Questionnaires, which synthesizes patient responses into scores for pain, mental health, self-image, functioning, satisfaction, and total QOL. Patients completed surveys throughout the preoperative and postoperative periods.

Results: Among 263 patients who underwent PSF, 188 (71%) were publicly insured and 75 (29%) were privately insured. Between groups, significant differences were noted for race (P<0.001) and ethnicity (P = 0.01), but no other demographic or preoperative characteristics. Publicly insured patients reported significantly worse preoperative scores for function (3.85 vs 4.11, P = 0.008), pain (3.88 vs 4.31, P = 0.001), self-image (3.18 vs 3.51, P = 0.003), mental health (3.81 vs 4.21, P = 0.002), and total QOL (3.63 vs 3.97, P<0.001). Postoperatively, publicly insured patients continued to report worse scores for pain at six weeks (3.73 vs 3.98, P = 0.03) and six months (4.16 vs 4.40, P = 0.04), and worse mental health scores at six weeks (3.97 vs 4.23, P = 0.03), six months (4.06 vs 4.30, P = 0.05), and one year (4.04 vs 4.36, P = 0.03).

Conclusions: Publicly insured patients were found to have worse QOL metrics in nearly all domains prior to surgery and for pain and mental health after surgery. Equalization of function, self-image, and total QOL metrics postoperatively suggests that access to appropriate surgical interventions may help mitigate some, but not all, of these preoperative differences.

This study provides Level III evidence, based on a retrospective cohort design.

## Introduction

In recent years, there has been an increasing emphasis on understanding the association between social determinants of health and pediatric orthopedic conditions, with the goal of achieving more equitable care [1]. Compared to privately insured counterparts, pediatric patients with public insurance have been shown to have less access to and longer wait times for specialty care, including orthopedics [1,2]. Adolescent idiopathic scoliosis (AIS) is the most common pediatric spinal condition and affects a diverse patient population. Several studies have examined factors contributing to health disparities in patients with AIS.

Publicly insured patients with AIS typically present with larger Cobb angles, increasing the likelihood of surgical intervention [3]. These patients also experience longer wait times for surgery and longer hospitalizations [3-5]. Insurance status, neighborhood opportunity as measured by the Childhood Opportunity Index (COI), and race are closely interrelated social determinants of health. Patients with public insurance, lower COI, and Black race have been shown to present with larger Cobb angles at initial evaluation [6,7].

Yet, there remains conflicting evidence in the literature regarding the influence of insurance status and broader socioeconomic factors on AIS presentation and outcomes. With respect to curve magnitude, several studies reported no significant differences in Cobb angle based on insurance status [2,7-9], and Nezwek et al. similarly found no correlation between the area deprivation index (ADI) and scoliosis severity [10]. Regarding treatment, some studies have shown that insurance status does not impact initial recommendation or surgical wait time [2,3]. In terms of postoperative outcomes, despite differences in comorbidities, length of stay, hospital costs, and number of vertebral fusions between publicly and privately insured patients, no significant difference in postoperative complications was observed [5]. Other studies have highlighted disparities related to both race and socioeconomic indices. For example, White patients were more likely to undergo surgical treatment compared to non-White patients [11]. Similarly, patients with lower COI scores presented with curve angles comparable to those of patients with higher COI scores; however, they were found to have a decreased risk of surgical complications [12].

Although investigation into healthcare disparities in patients with AIS continues to expand, we do not yet know how insurance type is associated with patient quality of life (QOL) in this patient population. The objective of this study is to investigate the association between patient QOL and insurance type in adolescents requiring surgical management for AIS. To analyze QOL, we used the data from the Scoliosis Research Society’s (SRS) SRS-30 and SRS-22r questionnaires, which have been widely used and validated by several studies [13,14]. Characterizing the impact of social determinants of health, such as health insurance, will help continue to identify and address disparities in QOL for the pediatric orthopedic patient population. 

## Materials and methods

Study population and setting

This retrospective cohort study was conducted among AIS-PSF patients who were surgically managed at a large, urban tertiary children’s hospital between 2017 and 2022. Inclusion criteria encompassed all AIS patients undergoing posterior spinal fusion (PSF) who completed the SRS surveys preoperatively and postoperatively. Exclusion criteria included patients with incomplete survey data or those undergoing revision surgeries. Incomplete survey data were defined as failure to complete at least one entire domain of the SRS instrument; partial omissions within a domain were retained if scoring could still be calculated. Surgical indications were consistent across insurance groups and based on standardized institutional criteria. This study was approved by the Institutional Review Board of the authors’ affiliated institution. Written informed consent was obtained from parents/guardians with patient assent for children aged 7-12 years. For patients aged 13-17 years, both the patient and parent/guardian signed the consent form. Patients ≥18 years provided their own consent. Patients who transitioned into a new age category during follow-up were re-consented in accordance with protocol requirements.

Measures of patient-reported outcomes

The primary independent variable of interest was insurance type, classified as public or private based on the insurance plan at the time of PSF. For patient-reported outcomes, we used the Scoliosis Research Society’s SRS-30 and SRS-22r questionnaires, which generate composite scores for pain, mental health, self-image, function, satisfaction, and overall quality of life (QOL), each on a 5-point scale (lower scores indicate worse outcomes). Questionnaires were administered during routine clinic visits through our institution’s Epic electronic health record system. Patients completed the surveys electronically on Epic-linked devices, with research coordinators present to provide assistance if needed. Responses were entered directly into the medical record and later extracted for analysis. The SRS-30 was used for patients enrolled prior to 2021. In 2021, our institution transitioned to the SRS-22r in accordance with updated guidance from the Scoliosis Research Society, which removed the SRS-23, SRS-24, and SRS-30 from its website and recommended universal adoption of the SRS-22r as the most recent validated version of the instrument.

Both the SRS-30 and SRS-22r are organized into the same five domains (Function/Activity, Pain, Self-Image/Appearance, Mental Health, and Satisfaction with Management). The SRS-30 consists of 30 items distributed across Function/Activity (seven items), Pain (six items), Self-Image/Appearance (nine items), Mental Health (five items), and Satisfaction (three items). The SRS-22r contains 22 items, including five items each for Function, Pain, Self-Image, and Mental Health, and two items for Satisfaction. Each question is scored from 1 (worst) to 5 (best). When summed, the SRS-22r yields a minimum possible score of 22 and a maximum of 110, while the SRS-30 yields a minimum of 23 and a maximum of 115 for the core items, with postoperative satisfaction items increasing the maximum to 150. For this analysis, we did not use summed total scores given the use of both instruments; instead, domain means were calculated for each of the five domains, and a total QOL score was calculated as the mean of all domains, ensuring comparability across both questionnaires. Domain scores were therefore reported as mean values on a 0-5 scale, regardless of instrument.

For Spanish-speaking patients, the Spanish version of the SRS-22r was used, which evolved from the validated Spanish translation of the SRS-22 [15,16]. Full English versions of the SRS-22r and SRS-30 questionnaires are included in Appendix A and Appendix B, respectively. The validated Spanish version of the SRS-22r is also provided in Appendix A. Permission to use the SRS-30 and SRS-22r was confirmed directly by the Scoliosis Research Society, which states that these questionnaires are free to use without licensing or additional approval. 

Statistical analysis 

Univariate analyses were performed to compare demographic and clinical variables between publicly and privately insured patients. Normality of continuous variables (age, BMI, baseline Cobb angle, follow-up Cobb angle, and other continuous measures) was assessed using the Shapiro-Wilk test and visual inspection of histograms and Q-Q plots. Normally distributed variables are presented as mean ± standard deviation (SD) and compared using independent-samples t-tests. For variables that did not meet normality assumptions (e.g., BMI, time between initial visit and surgery), data are presented as median (interquartile range, IQR) and compared using the Wilcoxon rank-sum test (Mann-Whitney U), with z-scores reported to reflect standardized test statistics.

Categorical variables (sex, race, ethnicity, insurance status, deformity type, treatment type, etc.) are summarized as counts and percentages and compared using Chi-square tests; Fisher’s exact test was applied when expected cell counts were <5.

Statistical significance was defined as a two-sided p<0.05 for all comparisons. All analyses were performed using Stata (Version 17.0, StataCorp, College Station, TX). A post hoc power analysis was conducted using G*Power (version 3.1, Heinrich-Heine-Universität Düsseldorf, Düsseldorf, Germany). For the preoperative total QOL comparison (n = 132 vs 56), the achieved power to detect a moderate effect size (d = 0.5) with a two-tailed Wilcoxon-Mann-Whitney test was approximately 84% at α = 0.05.

## Results

Among 263 AIS patients who were surgically managed between 2017 and 2022 at our hospital, 188 (71%) were publicly insured and 75 (29%) were privately insured. Significant racial and ethnic differences were observed between groups. Among publicly insured patients, 29.8% were Black and 36.2% were Hispanic, compared to 38.7% Black and 57.3% Hispanic in the privately insured group (Table 1).

**Table 1:** Pre-operative Characteristics by Insurance Type Gender, race, and initial recommendation were compared using chi-square tests; age at initial visit, initial Cobb angle, age at surgery, and Cobb angle at surgery were compared using Student’s t-tests; and BMI percentile and time between initial visit and surgery were compared using Wilcoxon rank-sum tests. SD: standard deviation, IQR: interquartile range

Parameter	N=188	N=75	Statistic	P-value
Gender, N (%)			0.003	0.97
Male	62 (33.0)	25 (33.3)		
Female	126 (67.0)	50 (66.7)		
Age at initial visit, average (SD)	13.8 (2.57)	13.4 (2.23)	-1.264	0.21
BMI percentile, median (IQR)	69 (43, 92)	74 (31, 95)	0.552	0.58
Race, N (%)				
White	5 (2.7)	11 (14.7)		
Black	56 (29.8)	29 (38.7)		
Other	94 (50.0)	27 (36.0)		
Unavailable	33 (17.6)	8 (10.7)		
Ethnicity, N (%)				
Hispanic	68 (36.2)	43 (57.3)		
Non-Hispanic	92 (48.9)	25 (33.3)		
Unavailable	28 (14.9)	7 (9.3)		
Initial Cobb angle, average (SD)	49.3 (12.8)	48.1 (13.2)	-0.676	0.50
Initial recommendation, N (%)			2.574	0.28
Observation	10 (5.4)	8 (10.7)		
Brace	61 (32.6)	21 (28.0)		
Surgery	116 (62.0)	46 (61.3)		
Age at surgery, average (SD)	15.3 (2.4)	14.8 (2.1)	-1.687	0.09
Cobb angle at surgery, average (SD)	58.2 (8.7)	60.4 (9.2)	1.806	0.07
Time between initial visit and surgery (months), median (IQR)	9.0 (4.0, 25.7)	11.9 (3.8, 27.3)	0.458	0.65

In contrast, there were no significant differences between groups in sex (public: 67.0% female vs private: 66.7% female, P = 0.97), age at initial visit (13.8 vs 13.4 years, P = 0.21), or BMI percentile (69 vs 74, P = 0.58). There were also no differences in initial treatment recommendation (observation, brace, or surgery, P = 0.28), Cobb angle at initial visit (49.3° vs 48.1°, P = 0.50), Cobb angle at surgery (58.2° vs 60.4°, P = 0.07), or time from initial presentation to surgery (9.0 vs 11.9 months, P = 0.65) (Table 1).

Preoperatively, publicly insured patients reported significantly worse quality-of-life scores compared to privately insured patients across multiple domains, including function (3.85 vs 4.11, P = 0.008), pain (3.88 vs 4.31, P = 0.001), self-image (3.18 vs 3.51, P = 0.003), mental health (3.81 vs 4.21, P = 0.002), and total QOL (3.63 vs 3.97, P < 0.001). Satisfaction scores did not differ significantly (3.22 vs 3.44, P = 0.17) (Table 2).

Postoperatively, disparities persisted in pain and mental health. Publicly insured patients reported lower pain scores at six weeks (3.73 vs 3.98, P = 0.03) and six months (4.16 vs 4.40, P = 0.04), while one-year pain scores trended lower but did not reach statistical significance (4.23 vs 4.50, P = 0.07). Mental health scores were lower among publicly insured patients at six weeks (3.97 vs 4.23, P = 0.03), six months (4.06 vs 4.30, P = 0.05), and one year (4.04 vs 4.36, P = 0.03). By contrast, there were no significant postoperative differences between groups in function, self-image, satisfaction, or total QOL at any time point (Table 2).

**Table 2:** Pre- and Post-operative Quality of Life Scores by Insurance Type SD: standard deviation Among 188 patients who completed pre-operative surveys (132 Public, 56 Private). Among 168 patients who completed 6-week post-operative surveys (112 Public, 56 Private). Among 137 patients who completed 6-month post-operative surveys (86 Public, 51 Private). Among 108 patients who completed 1-year post-operative surveys (70 Public, 38 Private).

Parameter	Public Insurance Mean (SD)	Private Insurance Mean (SD)	T Score	P-value
Pre-Operative				
Function	3.85 (0.63)	4.11 (0.52)	2.675	0.008
Pain	3.88 (0.86)	4.31 (0.69)	3.331	0.001
Self-Image	3.18 (0.68)	3.51 (0.66)	3.042	0.003
Mental Health	3.81 (0.83)	4.21 (0.68)	3.108	0.002
Satisfaction	3.22 (1.01)	3.44 (0.87)	1.381	0.17
Total	3.63 (0.55)	3.97 (0.48)	4.056	<0.001
6 Weeks Post-Operative				
Function	3.10 (0.67)	3.00 (0.56)	-0.929	0.35
Pain	3.73 (0.69)	3.98 (0.67)	2.170	0.03
Self-Image	4.09 (0.57)	4.08 (0.46)	-0.057	0.95
Mental Health	3.97 (0.75)	4.23 (0.60)	2.194	0.03
Satisfaction	4.33 (0.74)	4.43 (0.54)	0.907	0.37
Total	3.77 (0.44)	3.87 (0.37)	1.374	0.17
6 Months Post-Operative				
Function	3.56 (0.59)	3.64 (0.64)	0.678	0.50
Pain	4.16 (0.69)	4.40 (0.54)	2.091	0.04
Self-Image	4.09 (0.64)	4.20 (0.48)	1.115	0.27
Mental Health	4.06 (0.73)	4.30 (0.64)	1.956	0.05
Satisfaction	4.36 (0.73)	4.51 (0.56)	1.273	0.21
Total	4.00 (0.49)	4.15 (0.40)	1.798	0.07
1 Year Post-Operative				
Function	3.85 (0.74)	4.02 (0.42)	1.287	0.20
Pain	4.23 (0.85)	4.50 (0.44)	1.815	0.07
Self-Image	4.14 (0.62)	4.21 (0.49)	0.618	0.54
Mental Health	4.04 (0.80)	4.36 (0.66)	2.148	0.03
Satisfaction	4.39 (0.66)	4.49 (0.52)	0.739	0.46
Total	4.09 (0.59)	4.27 (0.36)	1.726	0.09

## Discussion

Our study found no significant differences between cohorts with respect to preoperative clinical characteristics, including initial Cobb angle, initial recommendation, and time to surgery. However, there were notable demographic differences. Patients with public insurance were more often categorized as "Other" race (50% vs 36%), whereas privately insured patients were more frequently White (15% vs 3%). Additionally, Hispanic ethnicity was more common among privately insured patients (57% vs 36%). Both race and ethnicity distributions differed significantly between groups.

Importantly, race and ethnicity themselves are known to influence scoliosis care and outcomes. Prior studies have shown that White patients are more likely to undergo surgical treatment compared to non-White patients [11], and that curve severity has been linked to public insurance in Black but not White patients [17]. These findings highlight that race and ethnicity may act as confounding factors in the relationship between insurance status and QOL. While we chose not to view race and ethnicity as independent variables in this analysis, our results should be interpreted within the broader context of overlapping social determinants of health. Future studies with larger, more diverse populations are needed to further disentangle the interplay between insurance status, race, ethnicity, and socioeconomic factors in AIS outcomes.

Our findings contribute to the mixed literature on the relationship between insurance status, disease progression, and management in AIS patients, aligning with studies that found no impact of insurance status on these metrics. For example, Goldstein et al. found no association between insurance status and initial Cobb angle or treatment recommendation, though publicly-insured patients experienced longer wait times for surgery [2]. Similarly, Pease et al. reported no link between curve severity and insurance type, race, or neighborhood disadvantage [7]. Russell et al. found that publicly insured patients had an average initial Cobb angle 2.9 degrees greater than privately insured patients, though the authors questioned clinical significance, as both cohorts were classified as “mild” scoliosis [8]. Similar results based on insurance status have been reported in several other studies [9,10]. 

Conversely, some studies indicate differences in these metrics based on insurance status and other socioeconomic markers. Patients with low COI and public insurance were found to have more severe curves and an increased likelihood of surgery [3,6]. However, other studies present contrary findings. Nuño et al. found that privately-insured and White patients were more likely to have surgery, and Linden et al. reported that patients with lower COI had decreased risk of postoperative complications [11,12]. 

Variability in results likely stems from varying study methodologies and differing patient populations. Complex interactions exist between race, ethnicity, socioeconomic status, insurance, and other factors. Heffernan et al. highlighted these interactions, showing that curve severity is linked to public insurance in Black patients, but not in White patients [17]. There is also a known variation of state screening policies for AIS in school-aged patients [12]. Our tertiary hospital serves a historically underserved, predominantly minority population with high rates of medical comorbidities [18]. 

Our study is the first to our knowledge to report on the relationship between insurance status and QOL metrics in AIS patients. Preoperatively, publicly insured patients had worse QOL scores across all domains, except for satisfaction, compared to privately insured patients. This disparity may be due to the direct impact of insurance type, the racial and ethnic differences seen between cohorts, and the role of insurance type as a proxy for other unmeasured differences between groups. Public insurance could affect QOL by limiting access to appointments and braces, as shown in prior studies [19]. However, this may be less likely in our cohort as we do not see a difference in initial recommendation or time to surgery. Insurance status is also regarded as a proxy for other socioeconomic markers such as income and education. Yet it is an imperfect proxy, as demonstrated by a study that reported misclassification rates of 14% and 23% for income and education, respectively, based on insurance status [20]. Other factors that may contribute include social determinants of health, such as transportation issues, barriers to compliance, language differences, and social support [19]. Such differences may contribute to poorer QOL before and during engagement with the healthcare system. 

In the postoperative period, patients with public insurance reported worse scores for pain and mental health, but comparable scores for function, self-image, satisfaction, and total QOL. This postoperative equalization of function, self-image, satisfaction, and total QOL suggests that surgical intervention and access to healthcare may have a leveling effect between public and private insurance patients. Access to appropriate surgical interventions may help mitigate some of these preoperative differences and highlight the need for additional interventions to address disparities when patients engage with healthcare. 

Despite equalization of many domains, publicly-insured patients still had persistently worse mental health and pain scores up to one year following PSF, as compared to privately-insured patients. Limited access to other specialties, such as mental health services, may be a factor. While many public insurances cover mental health services, barriers to access services exist. A 2022 article on the accessibility of mental health services in our hospital’s city identified the shortage of providers who accept public insurance as major barrier for patients who cannot pay out-of-pocket [21]. Hamersma and Ye found that public insurance expansions to cover pediatric mental health led to a significant decline in female mental health visits, although mental health outcomes did not decline [22]. This is clinically relevant considering AIS is more common in females, who make up two-thirds of our cohort. Pediatric orthopedic surgery teams have a unique opportunity to help address these disparities while patients are connected to the healthcare system, for example, by performing mental health screenings and directing patients to accessible services. 

This study demonstrates that AIS patients with public insurance may be at increased risk for persistent pain following PSF, despite similar clinical presentations and surgical indications. Similar relationships between pain and social deprivation have been observed in other pediatric orthopedic conditions. In children with upper extremity fractures, socially-deprived children reported poorer outcomes in function, mobility, pain, and peer relations, which did not improve after adequate fracture treatment [23,24]. To address these concerns, pediatric orthopedic teams should implement multimodal pain strategies and collaborate with interdisciplinary teams to improve outcomes for socially disadvantaged patients. Routine screening of high-risk patients, particularly those with public insurance, by social workers or mental health professionals in both the pre- and postoperative periods may help identify and manage psychosocial contributors to poor outcomes. Integrating therapy and mental health support throughout the surgical timeline, along with providing tailored preoperative education on surgery, recovery expectations, pain, and coping strategies, may improve patient preparedness and engagement. Further studies are warranted to better understand the mechanisms driving these disparities and to evaluate the effectiveness of targeted interventions. Potential strategies include integrating routine psychosocial support, expanding access to mental health services, and tailoring perioperative education for at-risk patients. Multicenter collaborations with standardized outcome collection across diverse geographic and clinical settings will be important to validate these findings, assess generalizability, and guide equitable, patient-centered care on a broader scale.

There are several limitations in this study. While racial and ethnic differences between cohorts may influence QOL scores, we chose not to view race and ethnicity as independent factors from insurance. Given that the majority of patients at our hospital and in this study are minorities, we believe these factors collectively contribute to our study’s context. Future research should explore the interplay of insurance status, race, and other socioeconomic factors in larger, more diverse cohorts to better understand their combined impact on QOL in AIS patients. Additionally, postoperative loss to follow-up introduces potential selection bias; at one year, 47% of public insurance patients and 32% of private insurance patients were lost. Pediatric patients with greater socioeconomic deprivation and public insurance are known to be at higher risk for loss to follow-up, reflecting a broader challenge in conducting long-term research in this population [25-27]. Another limitation is our use of both the SRS-30 and SRS-22r questionnaires, reflecting the transition in recommended instruments over the study period [28,29]. Although the SRS-22r was derived from the SRS-30, we analyzed mean domain scores (function, pain, self-image, mental health, and satisfaction) rather than raw item totals, which enhances comparability across instruments. Minor differences in item composition may introduce measurement non-equivalence, but the stability of our findings across domains suggests this is unlikely to have materially altered our conclusions. Furthermore, a systematic review in 2021 found that no AIS patient-reported outcome instrument, including the SRS tools, fully met recommended criteria [30]. Nevertheless, given their widespread validation and use, the SRS-30 and SRS-22r remain valuable for assessing QOL in this population [13,14].

## Conclusions

In this retrospective cohort study of AIS patients undergoing posterior spinal fusion, we found that publicly insured patients reported significantly worse preoperative QOL metrics in nearly all domains and experienced persistent deficits in pain and mental health postoperatively. These disparities occurred despite similar surgical indications, treatment timelines, and Cobb angles, underscoring the influence of nonclinical factors on patient-reported outcomes. While function, self-image, and total QOL improved to match privately insured peers following surgery, the persistence of mental health and pain disparities suggests a need for targeted, multidisciplinary interventions. Addressing these domains through integrated psychosocial support, pain management strategies, and improved access to mental health services may help bridge the gap for publicly insured patients. Future studies should explore these interventions longitudinally across diverse care settings to guide equitable, patient-centered strategies in pediatric orthopedic surgery.

## Figures and Tables

**Figure 1:**
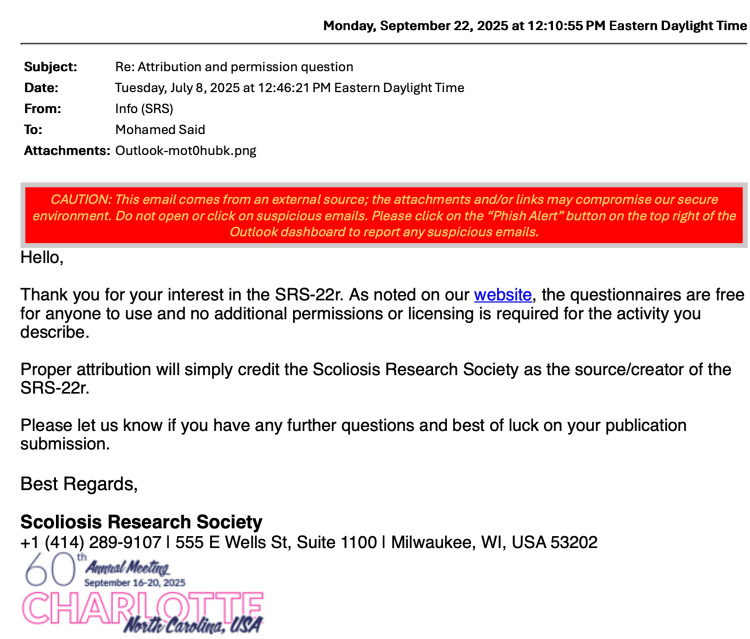
Permission confirmation email from the Scoliosis Research Society (SRS) for us of questionnaires Correspondence from the Scoliosis Research Society confirming that the SRS-30 and SRS-22r questionnaires are free to use without licensing or additional permission. Proper attribution should credit the Scoliosis Research Society as the source/creator. Abbreviations: SRS = Scoliosis Research Society.

## Appendices

Appendix A. Scoliosis Research Society-22 Revised Questionnaire 

**Table 3:** Scoliosis Research Society-22 Revised Patient Questionnaire The Scoliosis Research Society-22 Revised (SRS-22r) questionnaire is reproduced with permission from the Scoliosis Research Society (SRS). As publicly stated by the SRS and confirmed via direct correspondence, the questionnaire is free to use without licensing or additional permission. Proper attribution has been provided to the Scoliosis Research Society as the original source and creator of the instrument (Appendix C) [29]. The questionnaire has been reformatted into a bilingual tabular format, with original English items and their corresponding Spanish translations presented in separate rows to facilitate use in bilingual populations.

QUESTION.	ENGLISH VERSION	SPANISH VERSION
1	Which one of the following best describes the amount of pain you have experienced during the past 6 months? ☐ None ☐ Mild ☐ Moderate ☐Moderate to severe ☐ Severe	¿Cuánto dolor ha tenido en los últimos 6 meses? ☐ Ninguno ☐ Ligero ☐ Regular ☐Moderado ☐ Intenso
2	Which one of the following best describes the amount of pain you have experienced over the last month? ☐ None ☐ Mild ☐ Moderate ☐Moderate to severe ☐ Severe	¿Cuánto dolor ha tenido en el último mes? ☐ Ninguno ☐ Ligero ☐ Regular ☐Moderado ☐ Intenso
3	During the past 6 months have you been a very nervous person? ☐ None of the time ☐ A little of the time ☐ Some of the time ☐Most of the time ☐ All of the time	Durante los últimos 6 meses, ¿cuánto tiempo estuvo muy nervioso? ☐ Nunca ☐ Sólo alguna vez ☐ Algunas veces ☐ Casi siempre ☐ Siempre
4	If you had to spend the rest of your life with your back shape as it is right now, how would you feel about it? ☐ Very happy ☐ Somewhat happy ☐ Neither happy nor unhappy ☐Somewhat unhappy ☐ Very unhappy	Si tuviera que pasar el resto de su vida con la espalda como la tiene ahora, ¿cómo se sentiría? ☐ Muy contento ☐ Bastante contento ☐Ni contento ni descontento ☐ Bastante descontento ☐ Muy descontento
5	What is your current level of activity? ☐ Bedridden ☐ Primarily no activity ☐ Light labor and light sports ☐ Moderate labor and moderate sports ☐ Full activities without restriction	¿Cuál es su nivel de actividad actual? ☐ Permanentemente en cama ☐ No realiza prácticamente ninguna actividad ☐ Tareas ligeras y deportes ligeros ☐Tareas moderadas y deportes moderados ☐ Actividad completa
6	How do you look in clothes? ☐ Very good ☐ Good ☐ Fair ☐Bad ☐ Very bad	¿Cómo le queda la ropa? ☐ Muy bien ☐ Bien ☐ Aceptable ☐ Mal ☐ Muy mal
7	In the past 6 months have you felt so down in the dumps that nothing could cheer you up? ☐ Very often ☐ Often ☐Sometimes ☐ Rarely ☐ Never	Durante los últimos 6 meses, ¿se sintió tan bajo de moral que nada podía animarle? ☐ Siempre ☐ Casi siempre ☐ Algunas veces ☐ Sólo alguna vez ☐ Nunca
8	Do you experience back pain when at rest? ☐ Very often ☐ Often ☐Sometimes ☐ Rarely ☐ Never	¿Tiene dolor de espalda en reposo? ☐ Siempre ☐ Casi siempre ☐ Algunas veces ☐ Sólo alguna vez ☐ Nunca
9	What is your current level of work/school activity? ☐ 100% normal ☐ 75% normal ☐50% normal ☐ 25% normal ☐ 0% normal	¿Cuál es su nivel actual de actividad laboral o escolar? ☐ 100% de lo normal ☐ 75% de lo normal ☐ 50% de lo normal ☐ 25% de lo normal ☐ 0 % de lo normal
10	Which of the following best describes the appearance of your trunk; defined as the human body except for the head and extremities? ☐ Very good ☐ Good ☐ Fair ☐Poor ☐ Very Poor	¿Cómo describiría el aspecto de su cuerpo (sin tener en cuenta el de la cara y extremidades)? ☐ Muy bueno ☐ Bueno ☐ Regular ☐Malo ☐ Muy malo
11	Which one of the following best describes your pain medication use for back pain? ☐ None ☐ Non-narcotics weekly or less ☐ Non-narcotics daily ☐Narcotics weekly or less ☐Narcotics daily	¿Toma medicamentos para su espalda? ☐ Ninguno ☐ Calmantes suaves 1 a la semana o menos ☐ Calmantes suaves a diario ☐ Calmantes fuertes 1 a la semana o menos ☐ Calmantes fuertes a diario
12	Does your back limit your ability to do things around the house? ☐ Never ☐ Rarely ☐ Sometimes ☐ Often ☐ Very Often	¿Le limita la espalda la capacidad para realizar sus actividades habituales por casa? ☐ Nunca ☐ Sólo alguna vez ☐ Algunas veces ☐ Casi siempre ☐ Siempre
13	Have you felt calm and peaceful during the past 6 months? ☐ All of the time ☐ Most of the time ☐ Some of the time ☐ A little of the time ☐ None of the time	Durante los últimos 6 meses, ¿cuánto tiempo se sintió calmado y tranquilo? ☐ Siempre ☐ Casi siempre ☐ Algunas veces ☐ Sólo alguna vez ☐ Nunca
14	Do you feel that your back condition affects your personal relationships? ☐ None ☐ Slightly ☐ Mildly ☐Moderately ☐ Severely	¿Cree que el estado de su espalda influye en sus relaciones personales? ☐ Nada ☐ Un poco ☐ Regular ☐Bastante ☐ Mucho
15	Are you and/or your family experiencing financial difficulties because of your back? ☐ Severely ☐ Moderately ☐Mildly ☐ Slightly ☐ None	¿Ud. o su familia tienen dificultades económicas por su espalda? ☐ Mucho ☐ Bastante ☐ Regular ☐ Un poco ☐ Nada
16	In the past 6 months have you felt down hearted and blue? ☐ Never ☐ Rarely ☐ Sometimes ☐ Often ☐ Very often	En los últimos 6 meses, ¿se ha sentido desanimado y triste? ☐ Nunca ☐ Sólo alguna vez ☐ Algunas veces ☐ Casi siempre ☐ Siempre
17	In the last 3 months have you taken any days off of work, including household work, or school because of back pain? ☐ 0 days ☐ 1 day ☐ 2 days ☐ 3 days ☐ 4 or more days	En los últimos 3 meses, ¿cuántos días ha faltado al trabajo o al colegio debido al dolor de espalda? ☐ 0 ☐ 1 ☐ 2 ☐ 3 ☐ 4 ó más
18	Does your back condition limit your going out with friends/family? ☐ Never ☐ Rarely ☐ Sometimes ☐ Often ☐ Very often	¿Le dificulta la situación de su espalda salir de casa con sus amigos o su familia? ☐ Nunca ☐ Sólo alguna vez ☐ Algunas veces ☐ Casi siempre ☐ Siempre
19	Do you feel attractive with your current back condition? ☐ Yes, very ☐ Yes, somewhat ☐Neither ☐ No, not very much ☐No, not at all	¿Se siente atractivo/a con el estado actual de su espalda? ☐ Sí, mucho ☐ Sí, bastante ☐ Ni atractivo/a ni poco atractivo/a ☐ No, no demasiado ☐ En lo absoluto
20	Have you been a happy person during the past 6 months? ☐ None of the time ☐ A little of the time ☐ Some of the time ☐Most of the time ☐ All of the time	Durante los últimos 6 meses, ¿cuánto tiempo se sintió feliz? ☐ Nunca ☐ Sólo alguna vez ☐ Algunas veces ☐ Casi siempre ☐ Siempre
21	Are you satisfied with the results of your back management? ☐ Very satisfied ☐ Satisfied ☐Neither ☐ Unsatisfied ☐ Very unsatisfied	¿Está satisfecho con los resultados del tratamiento? ☐ Completamente satisfecho ☐ Bastante satisfecho ☐ Indiferente ☐ Bastante insatisfecho ☐ Completamente insatisfecho
22	Would you have the same management again if you had the same condition? ☐ Definitely yes ☐ Probably yes ☐ Not sure ☐ Probably not ☐Definitely not	¿Aceptaría el mismo tratamiento otra vez si estuviera en la misma situación? ☐ Sí, sin duda ☐ Probablemente sí ☐No estoy seguro/a ☐ Probablemente no ☐ No, sin duda

Appendix B. Scoliosis Research Society-30 Questionnaire 

**Table 4:** Scoliosis Research Society-30 Questionnaire The Scoliosis Research Society-30 (SRS-30) questionnaire is reproduced with permission from the Scoliosis Research Society. As publicly stated by the SRS and confirmed via direct correspondence, the questionnaire is free to use without licensing or additional permission (Appendix C). Proper attribution has been provided to the Scoliosis Research Society as the original source and creator of the instrument [29]. An official Spanish translation was not available. As of 2021, the SRS-30 has been removed from the official website, with the SRS now recommending universal adoption of the SRS-22r as the most recent and validated version of the instrument.

QUESTION.	ENGLISH VERSION
1	Which one of the following best describes the amount of pain you have experienced during the past 6 months? ☐ None ☐ Mild ☐ Moderate ☐ Moderate to severe ☐ Severe
2	Which one of the following best describes the amount of pain you have experienced over the last month? ☐ None ☐ Mild ☐ Moderate ☐ Moderate to severe ☐ Severe
3	During the past 6 months have you been a very nervous person? ☐ None of the time ☐ A little of the time ☐ Some of the time ☐ Most of the time ☐ All of the time
4	If you had to spend the rest of your life with your back shape as it is right now, how would you feel about it? ☐ Very happy ☐ Somewhat happy ☐ Neither happy nor unhappy ☐Somewhat unhappy ☐ Very unhappy
5	What is your current level of activity? ☐ Bedridden/wheelchair ☐ Primarily no activity ☐ Light labor, such as household chores ☐ Moderate manual labor and moderate sports ☐ Full activities without restriction
6	How do you look in clothes? ☐ Very good ☐ Good ☐ Fair ☐ Bad ☐ Very bad
7	In the past 6 months have you felt so down in the dumps that nothing could cheer you up? ☐ Very often ☐ Often ☐ Sometimes ☐ Rarely ☐ Never
8	Do you experience back pain when at rest? ☐ Very often ☐ Often ☐ Sometimes ☐ Rarely ☐ Never
9	What is your current level of work/school activity? ☐ 100% normal ☐ 75% normal ☐ 50% normal ☐ 25% normal ☐ 0% normal
10	Which of the following best describes the appearance of your trunk (defined as the human body except for the head and extremities)? ☐ Very good ☐ Good ☐ Fair ☐ Poor ☐ Very poor
11	Which one of the following best describes your medication usage for your back? Medication: _______ Usage (weekly or less or daily): _______ ☐ None ☐ Non-narcotics weekly or less (e.g., Tylenol, Ibuprofen) ☐ Non-narcotics daily ☐ Narcotics weekly or less (e.g., Percocet, Lorcet, Codeine, Darvocet) ☐ Narcotics daily ☐ Other
12	Does your back limit your ability to do things around the house? ☐ Never ☐ Rarely ☐ Sometimes ☐ Often ☐ Very often
13	Have you felt calm and peaceful during the past 6 months? ☐ All of the time ☐ Most of the time ☐ Some of the time ☐ A little of the time ☐ None of the time
14	Do you feel that your back condition affects your personal relationships? ☐ None ☐ Mildly ☐ Slightly ☐ Moderately ☐ Severely
15	Are you and/or your family experiencing financial difficulties because of your back? ☐ None ☐ Mildly ☐ Moderately ☐ Severely
16	In the past 6 months have you felt down-hearted and blue? ☐ Never ☐ Rarely ☐ Sometimes ☐ Often ☐ Very often
17	In the last 3 months have you taken any sick days from work/school due to back pain and, if so, how many? ☐ 0 ☐ 1 ☐ 2 ☐ 3 ☐ 4 or more
18	Do you go out more or less than your friends? ☐ Much more ☐ More ☐ Same ☐ Less ☐ Much less
19	Do you feel attractive with your current back condition? ☐ Yes, very ☐ Yes, somewhat ☐ Neither ☐ No, not very much ☐ No, not at all
20	Have you been a happy person during the past 6 months? ☐ None of the time ☐ A little of the time ☐ Some of the time ☐ Most of the time ☐ All of the time
21	Are you satisfied with the results of your back management? ☐ Very satisfied ☐ Satisfied ☐ Neither ☐ Unsatisfied ☐ Very unsatisfied
22	Would you have the same management again if you had the same condition? ☐ Definitely yes ☐ Probably yes ☐ Not sure ☐ Probably not ☐ Definitely not
23	On a scale of 1 to 9, with 1 being very low and 9 being extremely high, how would you rate your self-image? ☐ 1 ☐ 2 ☐ 3 ☐ 4 ☐ 5 ☐ 6 ☐ 7 ☐ 8 ☐ 9
24	Compared with before treatment, how do you feel you now look? ☐ Much better ☐ Better ☐ Same ☐ Worse ☐ Much worse
25	Has your back treatment changed your function and daily activity? ☐ Increased ☐ Not changed ☐ Decreased
26	Has your back treatment changed your ability to enjoy sports/hobbies? ☐ Increased ☐ Not changed ☐ Decreased
27	Has your back treatment _______ your back pain? ☐ Increased ☐ Not changed ☐ Decreased
28	Has your treatment changed your confidence in personal relationships with others? ☐ Increased ☐ Not changed ☐ Decreased
29	Has your treatment changed the way others view you? ☐ Much better ☐ Better ☐ Same ☐ Worse ☐ Much worse
30	Has your treatment changed your self-image? ☐ Increased ☐ Not changed ☐ Decreased

Appendix C. Permission confirmation email from the Scoliosis Research Society (SRS) for us of questionnaires 
